# Combining µXANES and µXRD mapping to analyse the heterogeneity in calcium carbonate granules excreted by the earthworm *Lumbricus terrestris*

**DOI:** 10.1107/S160057751303083X

**Published:** 2013-12-12

**Authors:** Loredana Brinza, Paul F. Schofield, Mark E. Hodson, Sophie Weller, Konstantin Ignatyev, Kalotina Geraki, Paul D. Quinn, J. Frederick W. Mosselmans

**Affiliations:** a Science Division, Diamond Light Source, Harwell Campus, Didcot, Oxon OX11 0DE, UK; b Mineral and Planetary Sciences Division, Natural History Museum, Cromwell Road, London SW7 5BD, UK; c Environment Department, University of York, York YO10 5DD, UK; d Department of Chemistry, University of Oxford, South Parks Road, Oxford OX1 3QR, UK

**Keywords:** full spectral XANES imaging, microfocus diffraction mapping, earthworm granules

## Abstract

The use of fluorescence full spectral micro-X-ray absorption near-edge structure (µXANES) mapping is becoming more widespread in the hard energy regime. This experimental method using the Ca *K*-edge combined with micro-X-ray diffraction (µXRD) mapping of the same sample has been enabled on beamline I18 at Diamond Light Source. This combined approach has been used to probe both long- and short-range order in calcium carbonate granules produced by the earthworm *Lumbricus terrestris*. In granules produced by earthworms cultured in a control artificial soil, calcite and vaterite are observed in the granules. However, granules produced by earthworms cultivated in the same artificial soil amended with 500 p.p.m. Mg also contain an aragonite. The two techniques, µXRD and µXANES, probe different sample volumes but there is good agreement in the phase maps produced.

## Abstract 2

A new experimental set-up enabling microfocus fluorescence XANES mapping and microfocus XRD mapping on the same sample at beamline I18 at Diamond Light Source is described. To demonstrate this set-up the heterogeneous mineralogy in calcium carbonate granules excreted by the earthworm *Lumbricus terrestris* has been analysed. Data analysis methods have been developed which enable µXRD and µXANES two-dimensional maps to be compared.

## 1. Introduction

Synchrotron microfocus X-ray imaging techniques have been applied across a range of sciences in the last 25 years (*e.g.* Sutton *et al.*, 1994 ▶; Manceau *et al.,* 2002*a* ▶; Fahrni, 2007 ▶; Lombi & Susini, 2009 ▶; Marcus, 2010 ▶; Fittschen & Falkenberg, 2011 ▶) since the advent of third-generation synchrotron sources with much lower emission characteristics. Thus far there have been two main ways in which micro-X-ray absorption spectroscopy (µXAS) has been acquired using microfocus optics. In the hard X-ray regimes single-spot µXANES or µEXAFS collection has been used, where a particular point, often specified from a micro-X-ray fluorescence (µXRF) map, is illuminated and the µXAS spectrum is collected by scanning the energy of the incoming X-ray beam. In the last 20 years, particularly in the soft X-ray region (<1 keV), *e.g.* at the C *K*-edge or 3*d* transition metal *L*-edges (Zhang *et al.*, 1994 ▶; Jacobsen *et al.*, 2000 ▶), large numbers of µXANES spectra from one sample have been produced by collecting a number of either transmission, electron yield or fluorescence maps at sequential energies (*e.g.* Paunesku *et al.*, 2006 ▶; Wan *et al.*, 2007 ▶) and then using software such as *Axis2000* (Hitchcock *et al.*, 2004 ▶) to stack the XRF maps and probe the energy dimension (Leung *et al.*, 2010 ▶). There are two experimental approaches to full spectral XANES imaging: the full-field approach, where the whole image at each energy is collected in one measurement, and the raster-scan approach with a focused beam, where each pixel of a map is collected individually.

Full spectrum mapping has recently been developed in the hard X-ray regime using an energy-dispersive set-up at the ESRF (Muñoz *et al.*, 2008 ▶), where the whole energy spectrum is collected in one shot at each pixel. In a similar manner to that used in the soft X-ray regime (*e.g.* Zhang *et al.*, 1994 ▶), full-field transmission hard X-ray XANES mapping has been demonstrated at the ESRF (Fayard *et al.*, 2013 ▶). Full-field fluorescence XANES mapping with a much larger pixel size has been demonstrated using a half-capillary lens to direct the emitted X-rays into a position-sensitive detector (Scharf *et al.*, 2011 ▶). On hard X-ray energy-scanning beamlines few full-fluorescence µXANES mapping experiments have been reported. Hard X-ray fluorescence XANES raster mapping has recently been demonstrated at the Australian Synchrotron (Etschmann *et al.*, 2010 ▶), and the XFM beamline at the Australian Synchrotron (Paterson *et al.*, 2011 ▶) is one of the few X-ray microprobe beamlines where such experiments are being conducted. This is partly explained by the fact that even with relatively short acquisition times per point (*e.g.* 0.05 s) the total acquisition time is still of the order of hours. Thus beamlines with large solid angle acceptance fluorescence detectors such as the MAIA detector (Kirkham *et al.*, 2010 ▶) have a substantial advantage. Oxidation-state mapping has been employed on hard X-ray microprobes (*e.g.* Pickering *et al.*, 2000 ▶; Berry *et al.*, 2013 ▶); while this is a proven technique for identification of different oxidation states (Sutton *et al.*, 1995 ▶), it does not necessarily differentiate different phases or species of the same oxidation state.

Diffraction mapping on X-ray microprobes, however, is a commonly applied technique. X-ray diffraction (XRD) on these facilities is particularly useful for phase identification within heterogeneous mineral samples, and when combined with µXRF the mineral species with which specific trace elements are associated can be identified (*e.g.* Rindby *et al.*, 1997 ▶; Tamura *et al.*, 2002 ▶; Manceau *et al.*, 2002*b* ▶). While X-ray diffraction gives a measure of long-range order in crystalline materials, XAS probes the short-range order that provides a complementarity that is increasingly being applied to studies of dynamic or heterogeneous systems.

A recent development on the I18 beamline at Diamond Light Source has been the introduction of full spectrum fluorescence XANES mapping (Noguchi *et al.*, 2013 ▶). We are unaware of any reports where both full spectral fluorescence µXANES mapping and µXRD mapping have been combined to investigate the same sample. This combination of techniques potentially allows the collection of a map of local speciation information on a minor elemental component, while the structural phase map is determined by µXRD. In this paper we report the two techniques being applied to a mineralogical study of biogenic calcium carbonate granules produced by an earthworm living in artificial soil (Darwin, 1881 ▶; Piearce, 1972 ▶; Lambkin *et al.*, 2011 ▶). In this example, the mineralogy is being probed by both techniques allowing a comparison of the two methodologies. We are attempting to understand the effects that the chemistry of the soil that the earthworm inhabits (and into which it secretes the granules) has on the mineralogy and composition of these granules; we have previously explored the incorporation in the granules of different metal ions such as Pb (Fraser *et al.*, 2011 ▶), Sr (Brinza *et al.*, 2013*a* ▶) and Zn (Brinza *et al.*, 2013*b* ▶). Previous reports of the calcium carbonate mineralogy of these granules suggest they comprise mainly calcite but can contain varying amounts of vaterite, aragonite and amorphous calcium carbonate (ACC) (Gago-Duport *et al.*, 2008 ▶; Lee *et al.*, 2008 ▶). There is substantial literature on the effect of Mg on calcium carbonate phases (*e.g.* Reddy & Nancollas, 1976 ▶; Aizenberg *et al.*, 2002 ▶). In inorganic seawater systems Mg has been found to favour the production of aragonite and Mg-calcite over calcite (Ries *et al.*, 2008 ▶), while in biogenic systems it is believed to stabilize ACC phases (Politi *et al.*, 2009 ▶). Isaure *et al.* (2010 ▶) have looked at the effect of Cd on Mg containing CaCO_3_ excreted by tobacco grains. Here we combine µXANES and µXRD mapping to examine three biogenic calcium carbonate granules; two of the granules were produced by earthworms inhabiting an Mg-amended soil, the other by an earthworm inhabiting a control artificial soil.

## 2. Experimental methods

### 2.1. Sample preparation

*Lumbricus terrestris* earthworms (The Recycleworks Ltd) were kept individually in 300 g (dry weight) of an amended artificial soil mixture at a moisture content of 36% of the dry soil mass and 289 K. The artificial soil comprised 10 wt% sieved peat (The Garden Centre Group Ltd), 20 wt% Polwhite B China Clay (Richard Baker Harrison Ltd) and 70 wt% ground silica sand (Richard Baker Harrison Ltd). For the experiment reported here, 7.5 g of powdered CaCO_3_ (Fisher Scientific UK) was added to each batch of 300 g of soil in order to obtain a soil pH at which the earthworms survived. To produce the Mg-amended soils, 1.286 g of MgCl_2_.6H_2_O (Fisher Scientific UK) in aqueous solution was added to each batch to result in a 500 p.p.m. Mg-containing soil by dry weight with a moisture content of 36% of the dry mass. For each soil mixture (*i.e.* control and Mg-amended artificial soil), five replicates were run. After 21 days the earthworms were extracted from the soil containers and placed into new batches of the same type of artificial soil for a further 21 days at 289 K. Then the earthworms were removed from the soil and the soil sieved through a 500 µm mesh sieve to obtain the calcium carbonate granules excreted by the earthworms during the experiment. The CaCO_3_ powder grain size is much smaller than the granules produced by the earthworms and no granules are found in soil batches which are left without earthworms, hence the granules studied are biogenic in origin.

Selected granules from the two experiments were set in an epoxy resin (Epo-Fix, Struers Inc.) and then polished down on both sides to a thickness of ∼50 µm. The granularity and porous nature of the samples meant that after thinning to ∼50 µm they were very fragile and had a tendency to fragment and/or fold after demounting from their silica slide substrate. Therefore, the µXANES mapping was performed first, while the granule slices remained on the silica slide, and the XRD mapping was performed second after the sample had been demounted from the silica slide. This was not our preferred approach; however, it did provide the greatest chance of success with these samples.

For µXANES mapping the granules remained on their silica slide substrates using adhesive. After µXANES mapping the granule slices were floated off the slide using acetone to provide a 50 µm-thick specimen for µXRD analysis which could be held over a hole in an aluminium sample holder using adhesive tape. Here results from the study of one typical granule recovered from the ‘control’ un-amended artificial soil and two typical granules recovered from the Mg-amended artificial soil are reported. A sample of speleothem aragonite from Makapansgat Valley, South Africa, was provided by Dr A. Finch (University of St Andrews), while calcite (Rodriguez-Blanco *et al.*, 2011 ▶), vaterite (Bots, 2012 ▶) and ACC standards (Rodriguez-Blanco *et al.*, 2011 ▶) were prepared by standard synthetic methods.

### 2.2. Beamline set-up

The experiments were performed at beamline I18 of Diamond Light Source (Mosselmans *et al.*, 2009 ▶). The beamline uses a cryogenically cooled Si(111) monochromator. The mechanically bent Kirkpatrick–Baez Si mirrors are equipped with a Rh stripe. The mean incident angle onto the mirrors was 3.5 mrad. The bare Si stripe of the vertically focusing mirror was used for the µXANES mapping at the Ca *K*-edge to provide harmonic rejection, while the Rh stripe was used for the µXRD mapping at 12.0 keV. A beam size on the samples of 4 µm × 4 µm was used. Each sample was held in a plane at 80° to the incident beam, with the fluorescence detector positioned normal to the beam direction. For the control granule collected from the un-amended soil a nine-element Ortec Ge C-train detector was used with STFC Xspress2 processing electronics, while for the granules collected from the Mg-amended soil a four-element Vortex ME4 Si drifts detector with Xmap processing electronics from SII-Nanotechnology was used. Both detectors produce data in a common HDF5 format which simplifies data processing. The 10° take-off geometry was used to substantially reduce any effect of self-absorption on the XANES data. Diffraction data were collected using a VHR-125 camera with 4032 × 2526 pixels (Photonic Sciences). Scatter from a polymethyl-methacrylate cover on the Pb beamstop was collected using an avalanche photodiode (FMB Oxford) to enable a gauge of the thickness of the sample at each point to be made. The µXRD data were calibrated by measuring a Si standard (NIST SRM 640B) at the sample position.

The control granule from the un-amended artificial soil was examined using µXANES and µXRD mapping. The µXANES mapping area examined was 400 µm × 300 µm in 5 µm horizontal and 20 µm vertical steps; the µXRD mapping was performed on an area of 300 µm × 200 µm with a step size of 10 µm in each direction. For the µXRD maps there was a collection time of 10 s per point. For µXANES mapping, 76 µXRF maps were collected at discrete energies from 4.02 to 4.14 keV in rastering mode, where the sample continually traverses across the beam with a data collection time of 0.2 s per pixel. X-ray mapping for each granule slice from the Mg-amended soil was performed in a similar way over a 400 µm × 100 µm region where the optical microscopy of the granule showed significant variation. For µXRD maps the pixel spacing used was 25 µm in each direction and the collection time per point was 10 s. For these granules the µXANES map of 486 pixels at 76 different energies had a total collection time of 170 min, while 85 pixel diffraction maps for the same area were collected in about 25 min.

XRD and XANES data at similar sample orientations were measured for the powdered calcium carbonate standards to aid the interpretation of the µXRD and µXANES maps. During the acquisition of the XRD patterns the sample was moved through the beam to obtain the most representative powder pattern.

### 2.3. Data analysis

The Ca *K*-edge XANES spectra recorded from synthetic standards were processed using the program *Athena* (Ravel & Newville, 2005 ▶). XANES mapping data were converted from the individual nexus XRF map files produced by the beamline into a format suitable for the program **Mantis** (Lerotic *et al.*, 2004 ▶) using a Python script. This script integrates the XRF spectra map data for the selected energy window then divides it by the I0 reading at each point, and if desired can normalize the XANES spectra. Normalizing the data gives better speciation cluster analysis, while the raw spectra give a concentration factor to the map analysis. This latter option enables regions with less or none of the element of interest to be easily separated in *Mantis*. In *Mantis* the XANES spectra can be examined using principal component analysis (PCA) and then application of cluster analysis shows the different spectral types associated with each region of the sample (Lerotic *et al.*, 2004 ▶). The cluster map output from *Mantis* can then be shaded with the intensity of the element of interest from one of the XRF maps so that the relative XRF intensity is also reflected in the final output. These spectra can be compared with standard spectra to determine the speciation they represent. *Mantis* gives a PCA scree plot from which the number of significant spectral components in the map could be determined, but it was found that the most reliable way of determining the minimum number of spectra that could be used to describe the sample was to repeatedly calculate a map with a set number of clusters. If the map continually changed it was assumed that there were too many clusters being used and local minima were being reached. The number of clusters was then reduced and the process repeated. When the cluster map did not change on repeated analysis, it was assumed that the number of clusters had reached a minimum. This method works well normally. *Mantis* allows individual point XANES scans to be plotted and in the case of one of the Mg maps a component could be seen in individual spectra from a small portion of the map that was not represented by a cluster. This effect has also been seen in the analysis of other XANES maps from the beamline. This highlights the importance in PCA analysis of this type that a check is made that all species are represented.

The XRD data were recorded in the form of TIFF files (Mosselmans *et al.*, 2009 ▶). In the XRD data analysis the detector–sample distance and beam centre were determined using the Nika plug-in (Ilavsky, 2012 ▶) in the program *Igor Pro* (version 6.22) (Wavemetrics Inc.) and then the images were calibrated and radially integrated using the program *Dawn* (http://www.dawnsci.org/home). The output from *Dawn* is a Nexus file containing the 1D spectrum at each point of the map. Using a Python script each XRD peak could be defined by a region of interest and the peak integrated using a background subtraction based on interpolating between the intensity at each edge of the window. The net intensity of the relevant diffraction peaks at each point in the map is written in a format that can be read into the program *PyMCA* (Solé *et al.*, 2007 ▶).

Any deviation in the sample from that of a perfect randomly oriented powder will induce changes in the relative intensities of the peaks in powder diffraction data. In µXRD such as that used in this study, crystallites with sizes that equated to (or were often larger than) the diffracting volume and with a subsequently limited number of orientations often resulted in XRD patterns that only contain some of the expected peaks for the relevant mineral phase. Hence a methodology was developed to identify points on the sample as either belonging to one of the expected mineral phases or not belonging to any of these phases. This involved integrating all the main calcium carbonate polymorph diffraction peak intensities in *PyMCA*. For calcite this included the diffraction peaks (104), (110), (113), (202), (116) and (300) (Graf, 1961 ▶); for vaterite the peaks (004), (110), (112), (114), (211), (300), (118) and (222) (Meyer, 1969 ▶); and for aragonite the peaks (002), (021), (031), (040), (111), (112), (113), (121), (200), (212) and (330) (de Villiers, 1971 ▶). The intensities were then plotted in *PyMCA* and, by examining the individual pattern associated with a point, a cut-off limit where a mineral phase was identifiable in a single point’s 1D XRD pattern was established for each of the three phases. Using this method each point was assigned to calcite, aragonite or vaterite or to a mixture or indeed to none of these phases. Thus a map was produced showing the distribution of phases in the sample.

## 3. Results

### 3.1. XANES mapping

**Figure 1:**
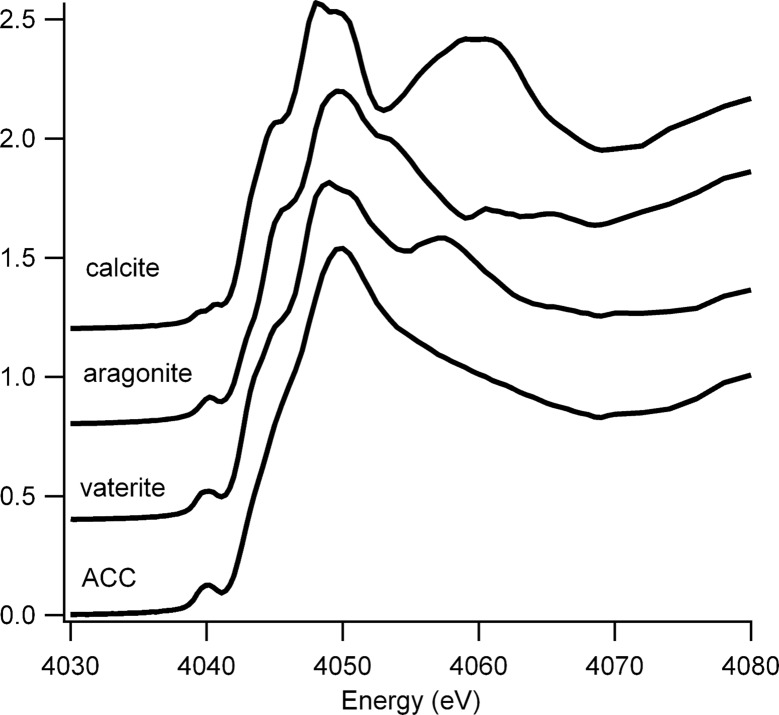
Ca *K*-edge XANES spectra of the calcium carbonate phases calcite, aragonite, vaterite and amorphous calcium carbonate (ACC).

Fig. 1 ▶ shows the Ca *K*-edge XANES spectra of the four calcium carbonate phases that, based upon our previous work (Fraser *et al.*, 2011 ▶; Brinza *et al.*, 2013*a* ▶,*b* ▶), we expected in the granules. The spectra are all significantly different (Levi-Kalisman *et al.*, 2000 ▶; Sarret *et al.*, 2007 ▶). In particular in the pre-edge region, calcite has two peaks while the other polymorphs have only one. All the spectra display a shoulder at ∼4045 eV, but the top of the white line is split into two for vaterite and calcite but not for aragonite or ACC. In the post-edge region vaterite has a second peak at 4058 eV, calcite has a broader peak at 4060 eV and aragonite has a shoulder on the trailing edge of the white line at 4054 eV and then two small peaks in the first depression at 4061 and 4065 eV. ACC shows a smooth decline from the white line to 4068 eV.

**Figure 2:**
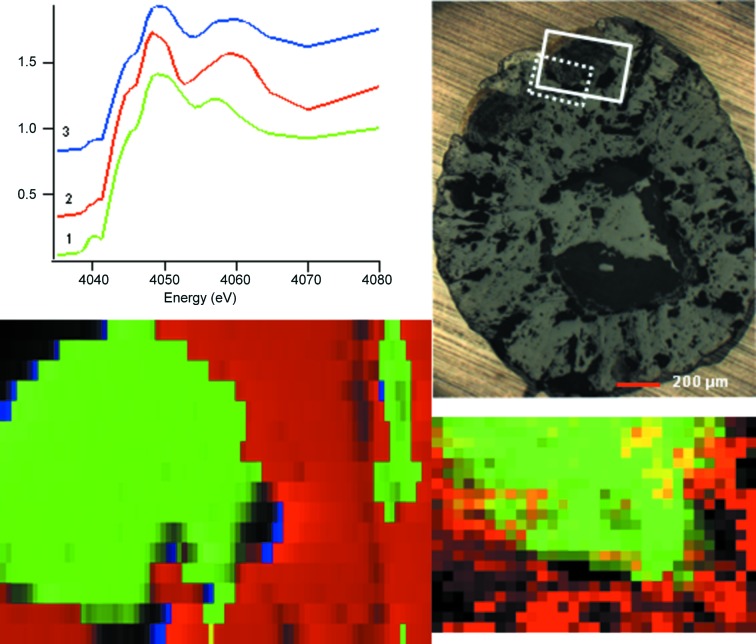
XANES cluster map (400 µm × 300 µm) (bottom left), cluster Ca *K*-edge XANES spectra (top left), optical microscope image (top right) and XRD map (300 µm × 200 µm) (bottom right) for a granule produced in un-amended artificial soil. The XANES map colours represent clusters denoted by the same colour in the XANES spectral image. In the optical micrograph the solid line denotes the area of the XANES map, while the dotted line denotes the area of XRD map. In the XRD map red represents calcite and green vaterite.

The XANES map for the granule from the control experiment (*i.e.* recovered from the un-amended soil) was deconvoluted in *Mantis* into three different clusters as shown in Fig. 2 ▶. Comparing the component spectra (Fig. 2 ▶) with our standards (Fig. 1 ▶), spectrum 1 is vaterite and spectrum 2 is calcite. Spectrum 3, which is only present in small amounts at the intersections between the vaterite and calcite regions, appears to be a mixture of these two phases in approximately a 1:2 vaterite:calcite ratio as determined by linear combination fitting. Hence the XANES mapping analysis reported here indicates that there are two phases, calcite and vaterite, in this area of the granule in agreement with bulk XRD measurements (Mosselmans *et al.*, 2013 ▶). No aragonite or ACC has been found in granules produced in the un-amended artificial soil (Mosselmans *et al.*, 2013 ▶).

**Figure 3:**
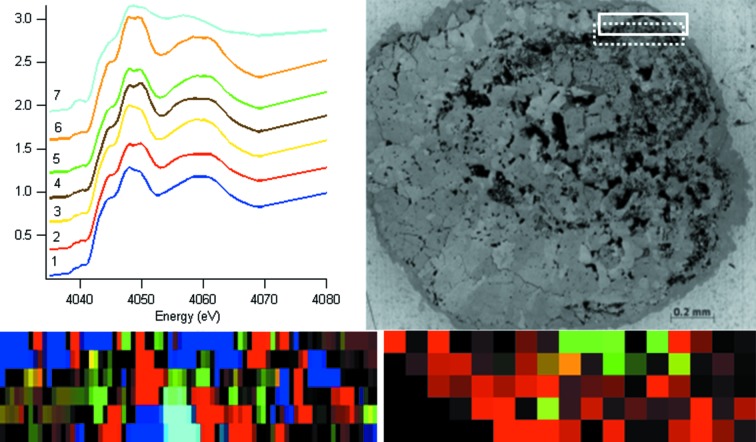
XANES cluster map (400 µm × 100 µm) (bottom left), cluster Ca *K*-edge XANES spectra (top left), optical microscope image (top right) and XRD map (400 µm × 100 µm) (bottom right) for granule Mg1 produced in 500 p.p.m. Mg-amended artificial soil. The XANES map colours represent clusters denoted by the same colour in the XANES spectral image. In the optical micrograph the solid line denotes the area of the XANES map, while the dotted line denotes the area of the XRD map. In the XRD map red represents calcite and green vaterite.

The two samples recovered from Mg-amended soil show different characteristics from each other. Mg1 shows three phases from cluster analysis; however, a small region of the map, that has vaterite-like spectra, is not represented well by any of the spectra from these three clusters. Indeed, for the whole XANES map to be well represented by cluster analysis, seven distinct phases are required (Fig. 3 ▶). When the XANES spectra from each of these clusters are examined in detail however (Fig. 3 ▶), spectra 1–6 are all representative of calcite, having two peaks in the pre-edge, two peaks on the white line and a further peak at 4060 eV. This suggests that these six different regions may just represent small calcite crystals that have different orientations with respect to the polarization of the X-ray beam. Indeed, we have previously shown that the single-crystal phases in earthworm-produced calcium carbonate granules are larger than the 4 µm X-ray beam footprint and significant differences may be seen in the ratio of the two peaks on the white line in the calcite XANES spectra, depending on the orientation of the crystals with respect to the beam (Brinza *et al.*, 2013*a* ▶). Here the other different regions may just represent different orientations of the calcite crystals that make up the area sampled. Spectrum 7 (in Fig. 3 ▶) is representative of vaterite.

**Figure 4:**
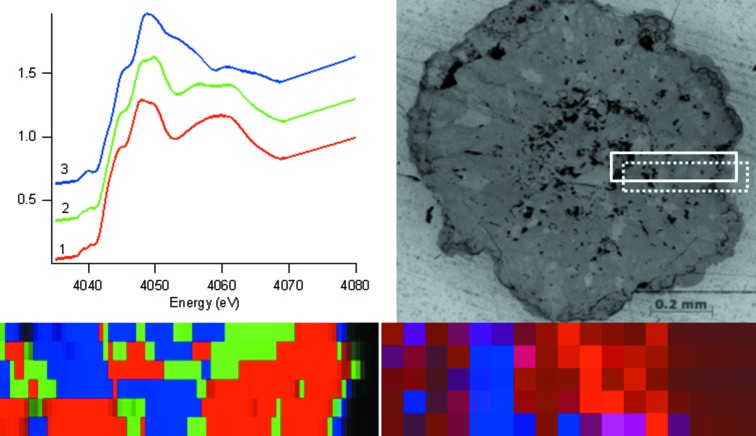
XANES cluster map (400 µm × 100 µm) (bottom left), cluster Ca *K*-edge XANES spectra (top left), optical microscope image (top right) and XRD map (400 µm × 100 µm) (bottom right) for granule Mg2 produced in 500 p.p.m. Mg-amended artificial soil. The XANES map colours represent clusters denoted by the same colour in the XANES spectral image. In the optical micrograph the solid line denotes the area of the XANES map, while the dotted line denotes the area of the XRD map. In the XRD map red represents calcite and blue aragonite.

The XANES cluster map for sample Mg2 (Fig. 4 ▶) shows only three phases. Spectrum 1 is similar to calcite with features similar to those described above, while spectrum 3 is similar to that of aragonite with the trailing shoulder on the white line and the two peaks in the first trough at 4061 and 4065 eV. Spectrum 2 is a mixture of calcite and aragonite in a roughly 6:4 ratio by linear combination fitting. Hence, in the region of this sample that has been studied, the XANES mapping indicates a mixture of aragonite and calcite.

### 3.2. XRD mapping

The granule from the control experiment showed two phases in its diffraction images, calcite and vaterite. The large area of vaterite in the XRD map corresponds to that seen in the XANES map (Fig. 2 ▶). The area of the sample mapped is slightly smaller than that used for the XANES mapping but it can be seen that the agreement between the two techniques is reasonable. The XANES imaging is sampling approximately one-sixth of the volume that is being probed by the XRD, hence the maps should not necessarily be identical. It was also noted from processing the data that some of the points have both calcite and vaterite peaks, hence the border around the large vaterite area is somewhat less defined than in the XANES map. This in turn suggests that the phase boundary between the calcite and the vaterite is not entirely parallel to the depth of the sample.

The XRD map from sample Mg1 (Fig. 3 ▶) shows calcite with a small amount of vaterite. Considering only the vaterite regions in the XANES and XRD maps of Fig. 3 ▶ (the calcite in the XANES cluster map being complicated by the crystal orientation effects described earlier) and accounting for the ∼50 µm vertical offset of the XRD map with respect to the XANES map, the positions of the vaterite component are commensurate and thus the maps agree. The XRD map from sample Mg2 (Fig. 4 ▶) shows aragonite and calcite in similar areas and amount to the XANES map for the same sample, taking into account the slightly different dimensions of the regions measured.

In these experiments the samples were demounted from their microscope slides after the XANES mapping in preparation for the transmission measurement of the XRD maps. This makes it difficult to ensure that the XRD maps precisely overlay the equivalent XANES map. However, despite the apparent differences in the positions of the phases observed in the XRD and XANES maps, the nature of the phases identified and their relative abundance is in good agreement.

## 4. Conclusions

At a 10° take-off angle the absorption length at 3.69 keV (Ca *K*α emission lines) on calcium carbonate is only around 5 µm. Hence the sampling volumes of the µXANES and µXRD maps are somewhat different. In spite of this the µXRD and µXANES maps are very similar and thus we conclude that in most cases the phase regions in the granules are bigger than the sampling volume of either technique. The observation that different replicates of granules from the same soil environment show slightly different mineral assemblages suggests that there is a natural variation imposed by the earthworm on the granule mineralogy. Thus in order to fully understand the effect of the soil chemical environment on the mineralogy of the granular output of *Lumbricus terrestris* a substantial sample set is being studied. Nevertheless, even with our relatively small sample set the identification of aragonite throughout the granules suggests that Mg induces the biogenic production and/or stabilization of aragonite in addition to calcite. This is perhaps in accord with the results of Ries *et al.* (2008 ▶), who found that Mg favours the production of aragonite in inorganic seawater systems. Biogenic aragonite was also found to be induced by the presence of Pb (Fraser *et al.*, 2011 ▶).

With high-intensity photon beams and larger area fluorescence detectors enabling faster hard X-ray fluorescence spectral µXANES mapping, we expect this technique to become more widespread. The use of direct photon-counting imaging detectors with fast readout is rapidly growing on synchrotron beamlines and thus more detailed faster µXRD mapping on a scale more commensurate with µXANES mapping is already feasible. Combining the two offers a local and long-range probe of structure and hence we expect that combined µXANES and µXRD mapping will become more widely applied tools in the analysis of heterogeneous crystalline materials.

We suggest that full spectral XANES mapping will also prove useful for reducing beam damage as the time taken for an 80 point spectrum is substantially less than one acquired by conventional means, especially if the monochromator does not have a continuous scanning capability. Continuous monochromator scanning may also cause some perturbation in the beam position on microfocus beamlines. Furthermore, because the energy order of acquiring the maps does not have to follow the scan energy linearly, maps at the most beam-sensitive energy (often in the edge region) may be acquired first, which is difficult to perform using a scanning monochromator.
